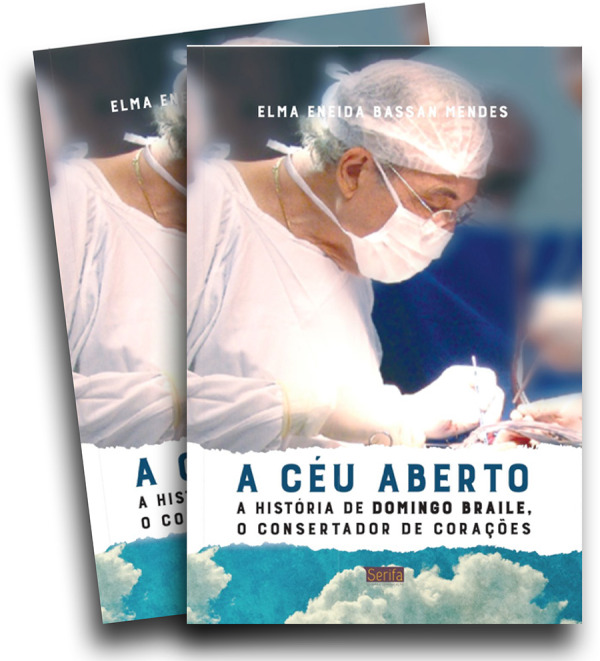# A Tribute to a Master: Professor Domingo M. Braile

**DOI:** 10.21470/1678-9741-1-2020-0606

**Published:** 2020

**Authors:** Walter J. Gomes, João Carlos F. Leal

**Affiliations:** 1 Discipline of Cardiovascular Surgery, Hospital São Paulo, Escola Paulista de Medicina, Universidade Federal de São Paulo - EPM-UNIFESP, São Paulo, SP, Brazil.; 2 Faculty of Medicine of São José do Rio Preto (FAMERP), São José do Rio Preto, SP, Brazil.

There are men who fight for a day and they are good. There are men who fight for a year and they are better. There are men who fight many years, and they are better still. But there are those who fight all their lives: These are the indispensable ones.

Bertolt Brecht

The excerpt from Bertold Brecht’s play *Die Mutter* fits well the character Professor Braile was. A singular man, a rare, perhaps a unique combination of a highly skilled heart surgeon, a devoted professor, an adroit scientist, a successful entrepreneur and the linchpin of a loving family.

An unchallenged leader in the field of cardiovascular surgery among us and the personification of the example to be followed by the younger generation in our country. His unparalleled and extraordinary achievements in life were made possible because of his energetic, persevering, and tireless personality, where every word out of his mouth resonated his real passion for the profession.

Nicknamed “the heart mender” in his biography, his endeavor allowed the country to master the production of a wide range of cardiovascular-related medical supplies, making possible the access to heart surgery through the public healthcare system to every patient in need, regardless of how little they earned or saved. And unquestionably contributing to make the Brazilian heart surgery powerful and recognized worldwide.

## EARLY LIFE

A son of an Italian doctor, Lino Braile, who immigrated from Italy to Brazil in 1929, and his wife Maria Neviane Braile, he was born in Nova Aliança, a small town in the Sao Paulo state countryside, on April 8, 1938.

As in his words, he had a strong influence from his father to pursue this profession, as well as motivated by his skill with machines and also inspired by his mentor, Professor Euryclides J. Zerbini.

His medical career began when he entered the Faculty of Medicine of the University of São Paulo in 1957, and soon joined the team of the Professor Zerbini, organizing the experimental workshop station of the Cardiac Surgery Service, as well as constructing and testing components of the earlier homemade prototypes of the heart-lung machine and artificial valves at the Department of Surgical Technique and Experimental Surgery of the Faculty of Medicine of the University of São Paulo. Afterwards, he was invited to the position of surgical assistant to the private surgical practice of Professor Zerbini, definitely contributing to his involvement in this specialty. At this time, a talented generation of young surgeons had gathered under the wing of Professor Zerbini, and eager to embrace the major challenge in the making of heart surgery in our country. Despite the appealing invitation to remain as a member of the attending surgical staff after graduating, he kept unbroken his original intention to return to his hometown, São José do Rio Preto.

## THE SURGEON

After returning to São José do Rio Preto in December 1962, he gathered a group of enthusiastic doctors and technicians, built his own heart-lung machine and a cardiology clinic from the scratch and performed the first cardiopulmonary bypass surgery at the Casa de Saúde Santa Helena in 1963.

In 1965, he started working at the Santa Casa de Misericórdia in São José do Rio Preto, where he established and headed the Cardiac Surgery Service until 1972. In 1967 led the group that created the Cardiovascular Diseases Institute (IMC) in São José do Rio Preto, where he stayed for 25 years working as the chief cardiac surgeon. In 1968 he was invited to be part of the clinical staff of the Infante D. Henrique Hospital of the Portuguese Beneficence Society of São José do Rio Preto, where he established the Cardiac Surgery Service. In that hospital, he also implemented the Intensive Care Service and the Medical Residency Service. In 1969 was elected clinical director, holding this position for 17 years. In 1991 founded the “Instituto Domingo Braile”, which performed from 1991 to 2007, under his responsibility and guidance, 6,340 cardiopulmonary bypass operations; 650 operations without cardiopulmonary bypass; and 5,760 pacemaker implants.

In his words “the first patient I operated on was a patient with a patent arterial duct, without cardiopulmonary bypass, but it was a victory, as a new era was inaugurated in São José do Rio Preto. After a few more patients operated on without cardiopulmonary bypass, we organized the correction of the first congenital defect with cardiopulmonary bypass. But not having adequate diagnostic equipment, what was thought to be an atrial septal defect, in reality it was a complex heart disease, which I was unable to correct, and the patient died. It was a big shock for me, and the team made up of general surgeons. Such failure was not enough to take our spirits away, even though it received a barrage of criticism. We went back to experimental surgery on dogs, so that the team could be trained for any event. We operate more than 30 dogs, (we had already operated another 30 before the first operation) The operations were always at night, because during daytime everyone had to work on each other’s hard routine. At the end of all this new training, we felt more secure and confident. In May 1963, we were able to operate on our second patient with cardiopulmonary bypass, a mixed mitral valve lesion, who survived and lived longer”.

In 1991, he created the Cardiac Surgery Service at the Hospital de Base of São José do Rio Preto, where 6,400 cardiac operations were performed until 2007. In total, personally or under his guidance, more than 30,000 cardiovascular operations were performed. Throughout his medical career, he created and helped the implementation of 21 medical services in several centers and hospitals, exercising the position of head of the cardiac surgery service and medical residency in seven of them, being the preceptor of 220 residents. His professional growth, always linked to scientific research, impelled him to create and develop techniques and products in the field of cardiovascular surgery. Since 1973 he was an enthusiast and researcher of myocardial protection techniques (mainly bloodbased cardioplegia), which resulted in the publication of several scientific papers and the standardization of his own technique. From 1988 onwards, he began developing a large experience with cardiomyoplasty, as an alternative to heart transplants, the results of which were recorded in his dissertation thesis presented at the Gama Filho University, in Rio de Janeiro in 1994.

Domingo M. Braile was invited to perform surgical demonstrations of operative techniques at several Brazilian medical centers and abroad: India, Japan, China, where he received the title of Honorary Professor at the University of Guiyang, in China.

A perfectionist surgeon repairing rheumatic diseased valves, we learned from him how to excel in valve reparative techniques.

## THE PROFESSOR

A founding member of the Faculty of Medicine of São José do Rio Preto (FAMERP), acted as an student director in charge for the admission and orientation of students from 1968 to 1973; head of the Department of Special Courses in the 1970 and 1971 biennium and head of the Cardiovascular Surgery Session of the Department of Surgery in 1971. He was professor at the Faculty of Medicine of São José do Rio Preto from 1968 to 1977; member of the Superior Council of the Hospital de Base of the Faculty of Medicine in São José do Rio Preto and head of the Cardiac Surgery Service until 1998. In 1990 he obtained the title of Doctor of Medicine (PhD) in the Postgraduate Course in Cardiovascular Surgery at Escola Paulista de Medicina, Federal University of São Paulo. He was professor in the Postgraduate Course in Health Sciences at the Faculty of Medicine of São José do Rio Preto from 1991 to 2012, teaching classes in the following disciplines: statistics applied to medicine, physiology of cardiopulmonary bypass and cardiac surgery methodology, and aerospace medicine among others. In 1994, was approved in the exam for qualification in medical teaching, a subarea of cardiovascular surgery at the Gama Filho University of Rio de Janeiro, with the thesis “Dynamic Cardiomyoplasty 10 Years: Critical Analysis and Personal Experience”, a subject he had dedicated his attention since 1986. He was also professor in the Graduation Course of the Faculty of Medicine of Catanduva, responsible for teaching cardiovascular and thoracic surgery from 1983 to 1994.

At one time, one of the most prestigious university of the country, the State University of Campinas (Unicamp), had its cardiovascular surgery department headless and inoperative, and a strong character was needed to fill the post and also face the many intrinsic challenges. Professor Braile was called upon and took the job, weekly travelling forth and back, he rebuilt the unity, aggregating the team, rehabilitating senior surgeons and training a new generation of heart surgeons, who marvelously replaced him at his departure. Though he retired as the head of the Cardiac Surgery Service, he held the position as a Senior Professor in the area of cardiac surgery at the Department of Surgery. From 1996 to 2012, he held the position of director and dean of postgraduate studies at the Faculty of Medicine of São José do Rio Preto. In his entire scientific career, he gave more than 900 lectures and conferences; presented more than 700 works (authorship and coauthorship); published more than 460 scientific articles; 25 book chapters and received 72 awards. He participated in more than 230 examining boards of Master, Doctorate, Livre-Docencia and public examinations, supervised 35 Dissertations and Theses. He was a member of 22 Editorial Boards and trained more than 200 cardiac surgery and cardiology residents, who work throughout Brazil, Latin America, and other countries. He was awarded with the title of Professor Emeritus at the Faculty of Medicine of São José do Rio Preto, where he was the Dean of *Stricto Sensu* Postgraduate Program and Director of Postgraduate Program.

## THE SCIENTIST

With an analytical and investigative mind, with problem-solving qualities, he was regularly looking for solutions in his daily affairs in his lab, operative room, his company, hospital etc. His lab and his company always had an open door for innovative or young surgeons to test new ideas, with no charges whatsoever.

At the beginning of his career, he had the opportunity to implant the earliest models of the caged-ball valves but sadly, these patients with valve disease were mostly from low socioeconomic and cultural status backgrounds and many of them living far away the nearest health facility, which made anticoagulation unfeasible and the use of mechanical prostheses impracticable. However, at the Hospital das Clínicas of the Faculty of Medicine of the University of São Paulo, professors Luiz B. Puig, Geraldo Verginelli and E. J. Zerbini had developed and were implanting the homologous dura mater bioprosthesis, preserved in glycerol. A remarkable achievement, no need of anticoagulants but after the good early results, late failures hindered enthusiasm, allied to the legal implications of tissue harvesting and low availability.

With this problem to be solved, he started to inquire about it, and reports of an artificial bioprosthetic built of chemically treated bovine pericardium raised his attention. A surgeon from England, Marian Ionescu, in 1971 had pioneered the construction and implantation of bovine pericardium valves, which offered a very low risk of embolization even in the absence of long term anticoagulation of the patients. He was enthusiastic about this proposal and in 1973 he implanted the first bovine pericardium valve developed by him and built in a dedicated laboratory. From this time onwards a series of modifications were made in order to improve the qualities and the performance of the pericardial xenograft. Of note, nearly 50 years later, the bovine pericardium is still the key material used for confection of conventional and transcateter valves. In 1977 it became a commercial product of his company, and a constant line of research in his life, originating, for example, his Doctoral Thesis, with the evaluation and follow-up of more than 575 patients over a period of 10 years.

Researcher of CNPq level 1B, he coordinated 10 innovation and development projects with 5 universities, with financial support of CNPq, FINEP and FAPESP. The result of the arduous journey of Research was winning the FINEP Award in 2011, 2012 and 2013, the FINEP National Innovation Award in 2013, the ABIMO INOVA Health Award in 2012 and 2013, the HEALTH Award in 2010, besides several awards of Best Work in conferences.

## THE EDITOR

A founding member of Brazilian Society of Cardiology’s Department of Cardiac Surgery in 1969, and in 1984 this Department was turned into the Brazilian Society of Cardiac Surgery, he was elected its President in 1992. He became the Editor-in-Chief of the Revista Brasileira de Cirurgia Cardiovascular, later renamed Brazilian Journal of Cardiovascular Surgery (BJCVS), from 2002 to his final days.

His fight and strive to bring the BJCVS to a higher standard were epic and worth recounting. With his commitment and dedication, the BJCVS submitted an application and was set to meet all scientific quality standard requirements for inclusion in MEDLINE, but the final word was protracted. He went right to Bethesda at the National Library of Medicine-PubMed, his suitcase and personal computer loaded with all the documents, and in a head-on talk with the boss, the aftermath being that the approval was soon issued. Later, going to London for a meeting at Thomsom Reuters, today Clarivate Analytics, Web of Science and finally being listed in the JCR (Journal of Citation Report) and having an Impact Factor. The transformation of the Revista Brasileira de Cirurgia Cardiovascular into the Brazilian Journal of Cardiovascular Surgery was a hallmark of the process of internationalization, expanding its visibility and importance.

## THE ENTREPRENEUR

Domingo Braile was a visionary and nationalistic doctor and his company, the Braile Biomédica is a reference in the field of manufacturing cardiovascular surgery supplies in Brazil.

In 1967 he founded the IMC (Cardiovascular Diseases Institute), along with other 5 colleagues, once again manifesting the idealization of a comprehensive service for the care of cardiac patients, serving thousands of patients and training hundreds of residents in cardiology and cardiac surgery. It has the status of a national technology research and development company, being a center of excellence for the Brazilian medical industry.

The company produces and supply a wide range of vital heart surgery components like heart-lung machines, membrane oxygenators and cardiopulmonary bypass sets. Under his direction, the production of biological heart valves and bovine pericardial grafts for cardiovascular operations took place from 1973 onwards.

In 1985 an external cardiac pacemaker and an esophageal stimulator were created for the study of arrhythmias and, in 1988, it developed a membrane oxygenator for the heart-lung circuit.

In 1998, he started developing products for the treatment of aortic diseases: endoprostheses, balloon catheter, vena cava filter and coils for embolization; and in the oncology field, endoprosthesis for the gastrointestinal tract and systems for intraperitoneal perfusion. In 2008, following the state of the art in minimally invasive surgery, he began researching and developing a biological valve for transcatheter implantation, the Inovare® Transcatheter Valve. This transcatheter valve won the FINEP National Innovation Award in 2013.

## THE MAN

An enthusiastic for aviation since childhood, his passion was flying his planes. At the beginning set up to fly gliders, then became a pilot shortly after high school and was qualified to fly solely by reference to instruments.

He owned and operated several planes throughout his life, granting him an amazing freedom to travel. Not few times stepped into his plane and set out to lecture or perform operation in remote places of the country. He was a founder member and president of the São José do Rio Preto Aeroclub for about 10 years, and vice president for 18 years.

And then, a sudden setback in his health and life. We were in Campinas at Unicamp for a defense of Doctoral Thesis of one of his students and Professor Braile was unusually coughing. One suggested a bronchoscopy, which he did, and a diagnosis of pharyngeal tumor was brought out. He went through a strenuous sequence of chemo and radiotherapy, but eventually he made a full recovery and soon was able to resume his professional activities. However, his body came out of this blow more fragilized, and he starts experiencing certain gait problems.

An enthusiastic storyteller, we could recall dozens of times where, late into the night, he was sharing his stories and the narratives, as we were falling asleep and he keeping on the stories.

Epic was also his fight to rescue his company, that in a turbulent time of global economic crisis, ran into financial trouble, on the brink of insolvency. Risking all his lifetime savings, sold and mortgaged his properties, some coming from family inheritance, along with heavy bank loans. Again, his temper prevailed and once more succeeded, the company was back on its feet shortly after.

In addition, he was a columnist for two newspapers in São José do Rio Preto with more than 550 articles published; and author of two books, a recollection of his articles: Millennium (2000) and Crônicas de um Médico do Sertão (Chronicles of a Doctor in the Hinterland) (2009). He won the 1st Place of Chronicles Category of the V Contest of Chronicles and Tales of the Brazilian Medical Association (AMB), with the chronicle “The Headlight”, in March 2017. In 2008 he became the Member of the number 11 Chair of Immortal Superior Council from São José do Rio Preto Academy of Letter and Culture. In 2013 he became Immortal from São Paulo Medicine Academy - chair number 48, whose patron is Prof. Dr. Dante Pazzanese.

He received the title of Honorary Member of Amazonas Medicine Academy in 2009, from Piauí Medicine Academy in 2013 and from Rio de Janeiro Medicine National Academy in 2014.

In addition, he received in 2016 the title of Doctor Honoris Causa from Piauí Federal University and also from São José do Rio Preto Technology University (FATEC) in 2009 and Guiyang University (China) in 2000.

In 1980 he received the Medal of Merit Santos Dumont, conferred by the Ministry of Aeronautics, for outstanding services to the Brazilian Aeronautics. Throughout his career he was awarded several titles and medals, among which the following stand out: title of commander of the Order of Benemerence in 1986 by the Government of Portugal and, in 2009, the Honorific Order of Ipiranga, conferred by the governor of São Paulo State, José Serra, for personal merits and services rendered to the State of São Paulo. Domingo Braile also received several awards, among them: Medalla a la Integración “Simón Bolivar” in 2001, Benedicto Montenegro Award granted by the Brazilian College of Surgeons - São Paulo Chapter in 2012, Grand Master of the Merit State Order Piauí Renaissance in 2016.

## THE FAMILY

Despite a busy multitasking man, he built a close-knit, cohesive family, a typical Italian-style family. Married to Maria Cecília Braga Braile, she was his co-pilot (occasionally the pilot) in the planes and in also in his life. In his words, “she was a partner of dreams, work, joy and many, many struggles and difficulties, with enormous love and patience”. From our outside view, she was additionally his bodyguard and lifelong protector.

His two daughters followed their father’s footsteps. Patricia, the elder one and a lawyer, is currently the president of the Braile Biomédica. Valeria, a cardiologist, runs the Braile Institute. His four grandchildren also followed his examples, three are doctors and the elder, Rafael, an engineer who is the CEO at Braile Biomédica, the third generation in the company.

He peacefully passed away on March 22, 2020, at the age of 81. In his final years, his body has paid a heavy toll for a life of hard work and countless time spent in the operative room. Several spinal disc herniation made painful his late life, underwent quite a few unsuccessful operations for fixing it and relieve pain. The saga of his life was narrated in the book “In the open sky - the story of Domingo Braile, the mender of hearts”.

Countless heart surgeons across the country, among them including ourselves, owns their career to Professor Braile’s support and mentorship. Every citizen in this country of 210 million people were made entitled the access to heart surgery, if in need. An example to be followed in these troubled times, where the rise of new “Brailes” will not only mend hearts, but also people and nations.

## Figures and Tables

**Prof. Dr. Domingo Marcolino Braile.**
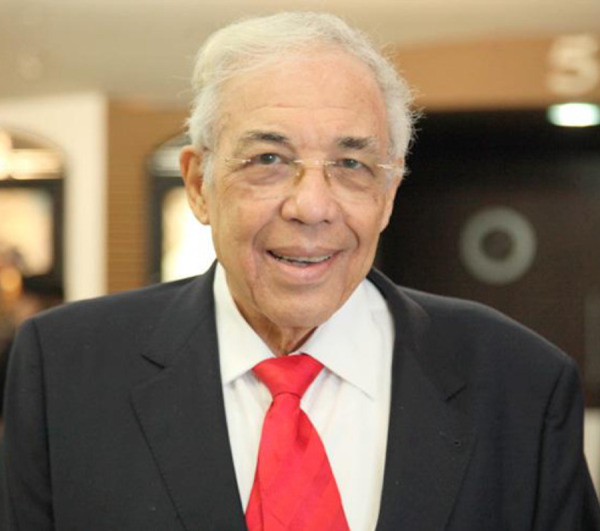


**Surgery with cardiopulmonary bypass at Hospital das Clínicas (SP), 1958. The team composed by Dagoberto Assunção, Rui Amaral, Prof. Zerbini, and Domingo Braile.**
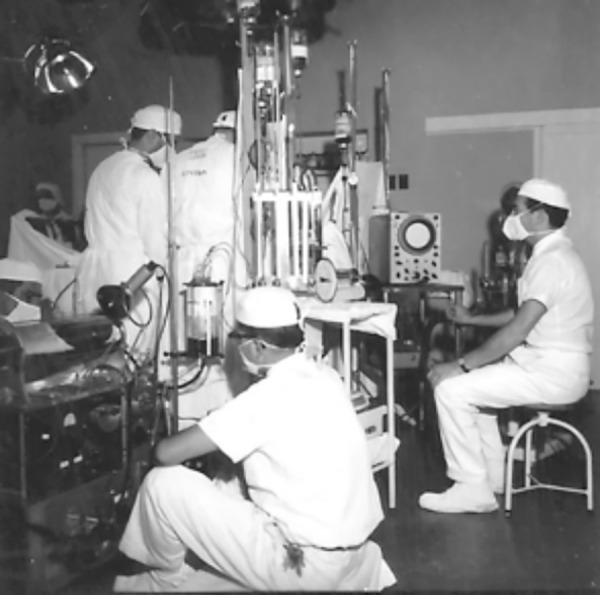


**Domingo Braile (center) during an experimental surgery.**
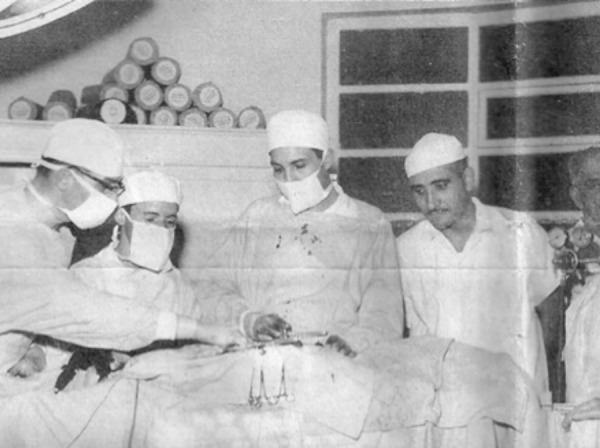


**Professor Braile and Professor Zerbini.**
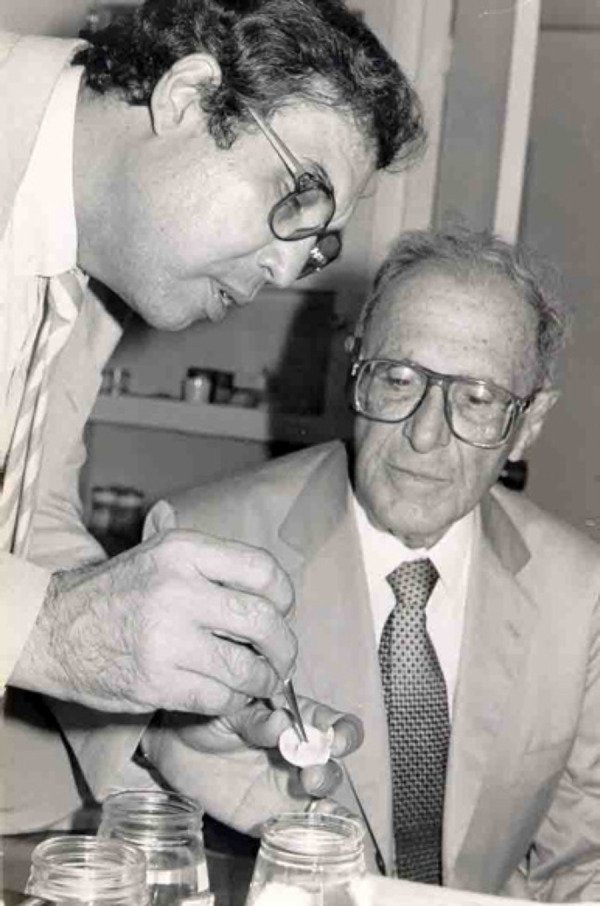


**Immortal from São Paulo Medicine Academy - chair number 48: Prof. Dr. Mário Santoro Junior (Ad Hoc Secretary of the São Paulo Medical Academy) and Prof. Dr. Affonso Renato Meira (President of the São Paulo Medical Academy), July 2013.**
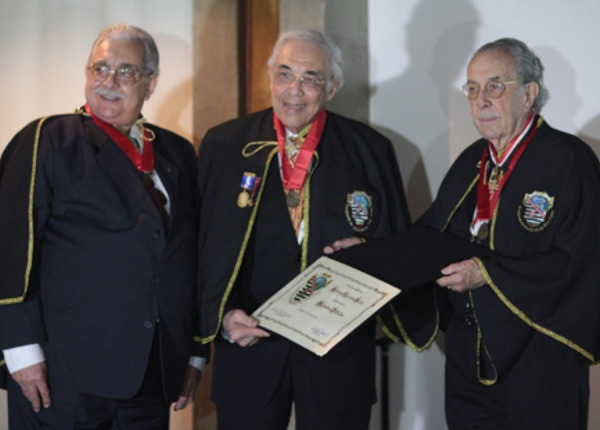


**The book cover of his biography.**